# Oncolytic Viruses as a Platform for the Treatment of Malignant Brain Tumors

**DOI:** 10.3390/ijms21207449

**Published:** 2020-10-09

**Authors:** Jana de Sostoa, Valérie Dutoit, Denis Migliorini

**Affiliations:** 1 Brain Tumor and Immune Cell Engineering Laboratory, Faculty of Medicine, University of Geneva, 1205 Geneva, Switzerland; jana.desostoa@unige.ch (J.d.S.); valerie.dutoit@unige.ch (V.D.); 2 Translational Research Center in Oncohaematology, Faculty of Medicine, University of Geneva, 1205 Geneva, Switzerland; 3 Swiss Cancer Center Léman, 1005 Lausanne, Switzerland; 4 Department of Oncology, Geneva University Hospital, 1211 Geneva, Switzerland

**Keywords:** oncolytic viruses, malignant brain tumors, clinical trials, cancer immunotherapy, combination therapy

## Abstract

Malignant brain tumors remain incurable diseases. Although much effort has been devoted to improving patient outcome, multiple factors such as the high tumor heterogeneity, the strong tumor-induced immunosuppressive microenvironment, and the low mutational burden make the treatment of these tumors especially challenging. Thus, novel therapeutic strategies are urgent. Oncolytic viruses (OVs) are biotherapeutics that have been selected or engineered to infect and selectively kill cancer cells. Increasingly, preclinical and clinical studies demonstrate the ability of OVs to recruit T cells and induce durable immune responses against both virus and tumor, transforming a “cold” tumor microenvironment into a “hot” environment. Besides promising clinical results as a monotherapy, OVs can be powerfully combined with other cancer therapies, helping to overcome critical barriers through the creation of synergistic effects in the fight against brain cancer. Although many questions remain to be answered to fully exploit the therapeutic potential of OVs, oncolytic virotherapy will clearly be part of future treatments for patients with malignant brain tumors.

## 1. Introduction

Malignant primary brain tumors represent one of the most difficult cancers to treat, leading to significant cancer-related morbidity and mortality in both children and adults. Gliomas are the most prevalent type of adult brain tumor, accounting for approximately 80% of malignant brain tumors. Glioblastomas (GBM) are endowed with the poorest overall survival, with <5% of patients surviving five years after diagnosis [1]. These tumors are usually treated with multimodal treatments comprising surgery, local radiotherapy, and systemic chemotherapy [2]. However, prognosis for patients remains very poor. Novel strategies are therefore needed to overcome the obstacles that prevent successful therapies in brain tumors, including the low tumor mutational burden [3,4,5], the high intratumoral heterogeneity [6], the tumor-induced immunosuppression [7,8], and the limited access to the tumors because of the blood-brain barrier (BBB) [9]. Oncolytic virotherapy can help to overcome some of these challenges, making it a promising new therapy for brain tumors.

Although the clinical concept of using viruses to treat cancer started in the mid-twentieth century [10], it was not until the 1990s, with the advent of recombinant DNA technology and virus genome engineering, that a new wave of virotherapy was initiated. Martuza and colleagues reported the first genetic modification that conferred a herpes simplex virus (HSV) the capacity to selectively replicate and destroy human GBM cells, with promising results both in vitro and in vivo [11]. In the following years, oncolytic viruses (OVs) gained attention for the treatments of those types of cancers. Indeed, several clinical trials evaluated the potential of a diverse group of OVs in patients with GBM and showed promising results [12,13,14,15]. OVs are therapeutically attractive, since they selectively infect and damage cancerous tissues while sparing normal tissue [16]. OV replication leads to the production of more virions, conferring them an uncommon feature in the field of pharmacology: a selective drug that amplifies within the target cells of the patient. Furthermore, OVs can be delivered systemically or locoregionally and, therefore, have the potential to act at both primary and metastatic tumor sites. Viral infection leads to tumor regression by the direct killing of tumor cells via replication-dependent induced cell death and by indirectly triggering an antitumor immune response via immunogenic cell death [17]. All types of immunogenic cell death, such as apoptosis, necrosis, and autophagic cell death, are characterized by the release of tumor-associated antigens (TAAs), damage-associated molecular patterns (DAMPs), viral pathogen-associated molecular patterns (PAMPs), and proinflammatory cytokines [18,19].

In this review, we discuss the current status of oncolytic virotherapy in malignant brain tumors, including the genetic modifications of OVs, recent progress in preclinical and clinical developments, combination therapy, and future prospects.

## 2. Oncolytic Viruses for Malignant Brain Tumors

Historically, two major achievements stirred up the oncolytic virotherapy field. First, in 1991, Martuza et al., reported prolonged survival of nude mice bearing intracranial U87 gliomas using genetically engineered HSV [11]. Second, in 2015, the US Food and Drug Administration (FDA) approved the granulocyte-macrophage colony-stimulating factor (GM-CSF)-expressing talimogene laherparepvec (T-VEC) HSV for the treatment of unresectable advanced melanoma [20,21,22]. Both Martuza’s work and the regulatory approval of T-VEC opened the field and generated substantial interest in the development of new OVs for cancer treatment. To date, a wide range of viruses have been investigated as potential cancer therapeutics for brain tumors, and the progress made with OVs in preclinical studies further encouraged the transition into clinical trials. In the next sections, we provide a description of the most common OVs and their genetic modifications, followed by an overview of the completed and ongoing clinical trials of OVs in brain tumors, and end with important advances in novel oncoviral therapies.

### 2.1. Tumor-Selective Viruses in the Clinic

A fundamental requirement in the development of OVs is to restrict their replication to tumor cells. OVs can be generally classified into two groups: naturally occurring or genetically engineered OVs. Naturally occurring OVs, such as parvoviruses, reoviruses, or Newcastle disease viruses (NDV), exert selective tumor replication without the need for further genetic modification. The second group, which includes adenoviruses (Ad), HSV, vaccinia viruses (VV), vesicular stomatitis viruses (VSV), and measles viruses (MV), is based on the genetic manipulation of viral genes to increase tumor selectivity and reduce virus pathogenicity (Table 1).

One strategy for selective replication is to delete viral genes that are necessary for efficient replication in normal cells but are dispensable in tumor cells. The ONYX-015 oncolytic Ad, approved as H101 in China in 2005 for the treatment of head and neck cancer [23], has a deletion of the *E1B-55K* gene, which is responsible for the binding and inactivation of the cell cycle regulator p53. This mutation renders the virus incapable of blocking p53 function, therefore restricting its replication in tumors that have already lost p53 [24]. DNX-2401 is a serotype 5 Ad (Ad5)-based OV that carries two genetic modifications that render it cancer-specific. The first one is a 24-base pair (bp) deletion in the *E1A* gene, which restricts viral replication to cells with deregulated pRB pathways, common in most GBM [25]. The second is the inclusion of an RGD-4C motif in the HI loop of the fiber, which redirects viral entry to cells expressing αvβ3- and αvβ5-integrins, enriched in glioma stem cells [26,27]. Moreover, this modification is especially relevant since several studies demonstrated the resistance of malignant glioma to adenoviral vectors, mainly attributed to low levels of coxsackie-adenovirus receptor, the primary receptor of Ad5, on brain tumor cells [28]. This virus has been widely studied in preclinical and clinical trials, demonstrating its safe profile and efficacy against gliomas [12,29]. Subsequently, a second-generation oncolytic Ad, DNX-2440 (or Delta-24-RGDOX), was designed [30,31]. This virus, which is currently being tested in a phase I clinical trial for GBM (NCT03714334), derives from DNX-2401 and expresses the *OX40L* gene. The expression of OX40L by Delta-24-RGDOX-infected tumor cells led to the enhanced proliferation of CD8+ T cells recognizing TAAs compared to its predecessor DNX-2401, demonstrating the potential of this virus to induce more potent T cell-mediated immunity within a tumor [30,31].

Several genetically engineered HSV have also been assessed in both preclinical and clinical studies for the treatment of brain tumors. Infected cell protein (ICP) 34.5 is a multifaceted protein of HSV involved in many aspects of viral pathogenesis, including neurovirulence [32,33]. Therefore, both copies of the *RL1* gene, which encode ICP34.5, were deleted in all HSV clinically evaluated in the brain, allowing replication in proliferating cells without affecting normal cells. After the generation of the ICP34.5-mutant HSV variant 1716 bearing these deletions, five more strains were evaluated in patients [34]. To further increase the safety of oncolytic HSV (oHSV), G207 OVs bear a *lacZ* insertion into the U_L_39 locus encoding the ribonucleotide reductase (RR) ICP6 protein [35]. It renders the viral RR, which is needed to provide deoxyribonucleotides for DNA synthesis in nondividing cells, nonfunctional. Actively dividing cancer cells encode homologs of RR that complement the loss of RR function. Later, G47Δ was generated by adding another deletion mutation to ICP47, a protein that inhibits peptide loading onto major histocompatibility complex (MHC) class I molecules on the surface of virus-infected cells. Thus, this mutation compromises evasion of the virus to specific immune recognition, aiming to enhance tumor cell killing via the increase of antitumor immune responses [36]. Another strategy that aims at the targeted expression of ICP34.5 uses tumor-specific promoters. One example is the rQNestin34.5 virus, in which the expression of ICP34.5 remains under control of the nestin-1 promoter, an intermediate filament protein upregulated in a high percentage of human glioma cells but not in normal astrocytes [37]. Finally, a human interleukin (IL)-12-expressing oHSV, designated M032, lacks both copies of the *RLI* gene but retains U_L_39 [38]. IL-12 is an immunostimulatory cytokine that has a direct antitumor activity, promotes interferon gamma (IFN-γ) production, and enhances immune effector functions [39].

PVSRIPO is a genetically modified nonpathogenic version of the Sabin type 1 poliovirus. CD155 is the entry site of this virus and is commonly overexpressed in GBM [40]. Moreover, high levels of CD155 have been found to play an important role in GBM cell invasion and intracerebral dispersion [40,41]. To suppress its intrinsic neurovirulence, the internal ribosomal entry site (IRES) of the poliovirus, which was discovered to be a critical neuropathogenesis determinant, was entirely exchanged by the IRES from human rhinovirus type 2 (HRV2) [42,43]. PVSRIPO has shown promising results in a phase I clinical trial for recurrent GBM [13].

TG6002 represents the only modified VV that has achieved clinical evaluation in patients with malignant brain tumors. This virus combines the deletion of the thymidine kinase (TK) gene (*J2R*) and the RR gene (*I4L*) to improve the tumor selectivity of its predecessor, the Copenhagen strain [44]. TK-deleted strains depend on the cellular pool of thymidine triphosphate and, thus, on the expression of cellular TK, which is highly expressed in cancer cells. As previously explained, tumor cells express homologs of the RR subunits. In addition, this virus expresses the *FCU1* gene, leading to the local conversion of the noncytotoxic prodrug 5-fluocytosine (5-FC) into 5-fluorouracil (5-FU), a widely used cancer chemotherapy. This strategy has also been used with the design of the nonlytic retroviral replicating vector (RRV) Toca 511 (Vocimagene amiretrorepvec), based on a modified murine leukemia virus. Toca 551 has been shown to elicit durable T cell-mediated antitumor immune responses in mouse glioma models [45,46].

Engineered MVs represent a promising oncolytic platform and are currently being evaluated in phase I trials. These viruses derive from the attenuated Edmonston strains of MVs that have safely been used for vaccinations since 1963. Afterwards, vaccine MVs demonstrated considerable oncolytic activity, mainly due to the overexpression of CD46, the virus entry site, on glioma cells [47]. MVs genetically engineered to express the human carcinoembryonic antigen (CEA) or the human sodium iodide symporter (NIS), both used to track viral gene expression in vivo, showed substantial antitumor activity against glioma cell lines and orthotopic xenografts and have been tested in patients with malignant brain tumors [47,48]. Although MV-CEA allowed the monitoring of viral replication through the detection of CEA in serum, it did not allow localization of the viral spread. In contrast, expression of the NIS protein allowed noninvasive monitoring of the viral infection both in vitro and in vivo using different isotopes [49]. Furthermore, MV-NIS has the potential to enhance virus-induced cytopathic effects through radiovirotherapy [48].

Among clinically relevant naturally occurring OVs, Reolysin (a reovirus), ParvOryx01 (a rat H-1 parvovirus), and NDVs have shown promising results in preclinical studies [50] and early phase clinical trials [51,52,53,54]. Reoviruses are nonpathogenic and have been reported to specifically replicate in cancer cells using the activated Ras signaling pathways to enhance proteolytic viral disassembly [55]. Parvovirus H-1, whose natural host is the rat, has demonstrated the induction of lysosome-dependent cell death in glioma cells, enabling to overcome glioma cell resistance of the proapoptosis cell death inducers, such as conventional chemotherapy agents like cisplatin or the soluble death ligands TRAIL [56]. Finally, NDV is an avian type I paramyxovirus that exhibits selective oncolytic properties, affecting also GBM [57]. MTH-68/H and NDV-HJU are live attenuated strains of NDV, MTH-68/H being a moderately pathogenic (mesogenic) strain and NDV-HJU an avirulent (lentogenic) strain [58].

### 2.2. Clinical Experience

To date, hundreds of patients have been treated using a wide range of viruses, doses, and routes of delivery. Consequently, some of the above-described viruses have reached phase I-III clinical trials as OVs to treat malignant brain cancers. Table 2 and Table 3 provide an outline of completed and ongoing clinical trials evaluating oncolytic virotherapy in the field of brain tumors, respectively.

Despite promising preclinical results with Ad, HSV, NDV, or reovirus, early clinical trials with these first-in-class viruses demonstrated the safety of the approach, but no significant objective clinical responses were observed (Table 3). However, a recent wave of trials deserves special attention.

Lang et al., recently reported the potential of DNX-2401 Ad5 in a phase I study for the treatment of recurrent glioma [12]. Thirty-seven patients were enrolled to receive a single intratumoral injection of DNX-2401 at escalating doses (group A, *n* = 25) or an intratumoral injection of the virus, followed 14 days later by tumor resection and a second dose of DNX-2401 into multiple sites in the wall of the resection cavity (group B, *n* = 12). The latter group allowed the characterization of the mode of action of the virus through analysis of the resected tumors. First, the expression of the viral E1A or hexon proteins and the detection of prominent inclusion bodies 14 days after treatment showed evidence of virus replication within the tumors in six of 11 patients. Surprisingly, although the immune analysis demonstrated increased numbers of CD4+ T cells as compared to pretreatment tumors, no changes in the expressions of PD-1, PD-L1, or IDO-1H were observed. Of note, signs of pseudoprogression (increase in lesion size as a consequence of treatment-induced immune cell infiltration) were observed in all three patients with complete responses (CR). This pattern, which is also observed with immune checkpoint inhibitors [59], has to be further explored but has been suggested to be associated with an improved clinical prognosis, as observed in this trial. When evaluating immune-related signals, a significant increase in DAMP markers was detected in glioma stem cells isolated from the surgical specimens of two patients, which is in-line with previous preclinical studies demonstrating the Ad induction of immunogenic cell death. Remarkably, and not frequently, no dose-limiting toxicity was observed, and, consequently, the maximum tolerated dose was not identified. In group A, 72% of patients had radiologic reductions in tumor size, and 20% of patients survived more than three years after treatment, with three CR (12%), whose progression-free survival (PFS) was over three years. The median overall survival (mOS) time was nine-and-a-half months. Within group B, patients were assessed only for survival and displayed a mOS of 13 months, with two patients (17%) surviving for two years. To confirm DNX-2401 as a clinical choice for GBM treatment, larger clinical trials comparing the virus to standard care should now be performed. However, this study provides valuable data to better understand the mechanism of action of the virus, suggesting direct oncolytic effects in human brain tumors, and provides evidence of antiglioma immune responses.

PVSRIPO is the protagonist of another worth-mentioning phase I clinical trial [13], which led to the breakthrough therapy designation for GBM by the U.S. FDA. A total of 61 patients with recurrent GBM were enrolled and received PVSRIPO intratumorally on a dose-escalation schedule. One dose-limiting toxic effect occurred at dose level 5, with intracranial hemorrhage immediately after catheter removal. However, the virus treatment was generally well-tolerated: 69% of all patients experienced grade 1 or 2 adverse events attributed to PVSRIPO. In the dose-expansion phase, 19% of patients had grade 3 or higher adverse effects, generally due to PVSRIPO-induced local inflammatory reactions. Importantly, the survival rate at 24 months was 21%, versus 4% in the historical controls, and was maintained at 36 months. The mOS among all patients was 12.5 months. Although this clinical study showed significant promise, it has to be noted that 37 of 61 (60.6%) patients received other immuno- or chemotherapy agent post-virus treatments. Out of 24 patients that received the monotherapy treatment, nine remained alive at the time of publication, with survival times over 15 months. Aside from the promising clinical benefits, it is still difficult to determine the contribution of PVSRIPO to these responses, mainly when combined with other treatments. Thus, further clinical trials with PVSRIPO as the single agent will be needed to answer whether the virus can infect and replicate in glioma tumor cells or if it can induce antitumor immune responses in patients. This is the aim of two ongoing phase Ib and II clinical trials testing PVSPIRO in children and adults (NCT03043391 and NCT02986178).

Another interesting dose-escalating clinical trial tested the ParvOryx01 H-1 parvovirus [51]. In contrast to other studies, recruited patients (*n* = 18) were assigned to two treatment arms differing in the mode of first virus application. In arm 1, ParvOryx was injected intratumorally, whereas, in arm 2, patients received five intravenous infusions on days 1 to 5. On day 10, all patients underwent tumor resection, and the virus was reinjected around the resection cavity. The ParvOryx treatment was shown to be safe, with no dose-limiting toxicity. One patient suffered an unexpected serious adverse reaction, but a further analysis could not relate this event to the virus treatment. Positive fluorescence in situ hybridization (FISH) signals of viral DNA were observed in 11 of the 12 tumors in the intratumoral injection cohort. Viral DNA was detected in the inoculation site and in catheter-distant tumor areas, confirming penetration into the tumor tissue. Importantly, virus RNA was revealed in four of the six tumors in the intravenous-treated patient cohort and viral DNA in three of six tumors, proving that ParvOryx can cross the BBB from blood to the tumor. Increased immune cell infiltrates were observed in virus-treated patients compared with the primary tumor. However, a primary tumor specimen was obtained from only one of the patients, making the comparison and interpretation of the histological and immunological findings more difficult. Tumor-infiltrating T cells were shown to express granzyme B and perforin, while the frequency of regulatory T (T-reg) cells was reduced. Last, nine of 12 tested patients were found to mount a significant T-cell response against viral antigens, and three of six patients showed a low but significant T-cell response to glioma antigens. Overall, PFS was 15.9 weeks, and mOS was 15.2 months. Clinical and scientific evidence obtained from this pilot study further supports the development of ParvOryx in trials aiming at including a higher number of patients in each arm and at obtaining tumor samples from all patients before and after virus treatment.

Reolysin recently showed encouraging results in a phase Ib clinical study [52]. In-line with Geletneky et al. [51], this study assessed the feasibility of using a systemic reovirus delivery in patients with high-grade glioma (HGG). Nine patients were treated with a single intravenous virus infusion, followed by surgical resection three to 17 days post-treatment. Grade 1 to 2 lymphopenia was observed in all patients and grade 3 to 4 in six patients. The detection of reovirus in resected tumors by immunohistochemistry (IHC), immunogold transmission electron microscopy, and in situ hybridization supported the delivery of intravenous-administered reovirus to brain tumors. Virus-treated patients displayed increased tumor-infiltrating CD8+ T cells as compared to untreated patients. CD68+ microglia/infiltrating macrophages were detected in higher numbers in tumors from reovirus-treated patients compared to controls. Finally, these tumors exhibited more intense staining for both PD-1 and PD-L1, a sign of a virus-induced antitumor immune response. The mOS was 15.4 months from the day of reovirus treatment. These results would justify a combination of reovirus infusion with subsequent anti-PD-1 treatment, as preclinically demonstrated in this study, with C57/BL6 mice implanted with GL261 glioma cells. Although it is still early to evaluate clinical outcomes, this study provides evidence that a systemic infusion of Reolysin can alter the tumor immune microenvironment. A new version of reovirus that expresses GM-CSF is currently being evaluated by systemic delivery in a phase I clinical trial for pediatric relapsed brain tumors (NCT02444546).

Last, a phase I dose-escalation clinical study [60] testing Toca 511 plus 5-FC in patients with recurrent HGG showed six durable complete responses out of 53 patients enrolled. All responders remained alive 33.9–52.2 months after virus administration. No virological, histological, and/or immunological analyses were performed. Unfortunately, the randomized phase III confirmatory study (NCT02414165), including 403 patients with anaplastic astrocytoma (AA) or GBM, failed to meet its primary endpoint, with an mOS of 11.1 months versus 12.2 months with the standard of care [61].

As previously mentioned, OV efficacy can be achieved by the direct cytotoxicity of tumor cells and/or by the activation of the immune system against the tumor. Taking that into account, objective evidence of the efficacy of oncolytic virotherapy in patients requires (1) the evaluation of OVs as a single-agent therapy, (2) analysis of the virus presence at the tumor site, (3) ability of the virus to infect and replicate in the tumor bulk, and (4) the detection of increased immune cell infiltration and immunostimulatory molecules in the tumor. A subsequent analysis of the specific immune response to both virus and tumor epitopes should be done. Monitoring these parameters will help to identify relevant OVs as therapeutic tools for patients with malignant brain tumors and could be used as engineering platforms for new immunotherapeutic therapies.

#### Viral Delivery

One of the most important steps of oncolytic virotherapy is the accurate, safe, and efficient delivery of the virus. Delivery to central nervous system (CNS) tumors is especially challenging. To date, multiple routes of delivery have been tested preclinically, including intrathecal, intravascular, intracerebral, and intratumoral deliveries. However, viral delivery in clinical studies has been mainly limited to direct intralesional injection of the OVs (Table 2 and Table 3). Intratumoral administration is usually chosen for safety reasons, to minimize the impact of preexisting circulating antibodies and to overcome the low CNS penetration of the virus across the BBB [72]. As mentioned above, recent studies have shown the feasibility of systemic virus delivery for CNS tumors, demonstrated by viral detection at the tumor site and the activation of immune responses [51,52]. In fact, it is often stated that an ideal OV should be systemically injectable, as this is less invasive, and should be able to reach both primary and metastatic tumors [73]. An example of that is the case of the approved T-VEC OV, in which, despite intratumoral injection, uninjected skin lesions and, occasionally, even visceral metastases displayed regression, likely due to the trafficking of tumor-specific T cells elicited by the virus [22,74]. Nevertheless, the metastatic spread of GBM outside of CNS is uncommon, with the incidence of extraneural metastasis reported at 0.2% [75]. With this low frequency, intravenous delivery would not be useful for metastases, but may improve virus distribution and spread within the tumor. Thus, the best delivery strategy depends on (1) whether the tumor is surgically accessible and intratumoral infusion is feasible, (2) whether the virus can cross the BBB and whether it is neutralized by the immune system, (3) the desired dose of administration, and (4) the clinical endpoint. Although intratumoral delivery looks so far as the favorite choice for brain tumors, there are other possible approaches. To achieve optimal antitumor responses, answering these questions will be critical.

### 2.3. Novel Potential Therapeutic Agents

Newly engineered OVs have been developed with the aim to improve the efficacy of brain cancer treatments, but they have not yet been evaluated in clinical trials. The following preclinical studies evaluated the safety and efficacy of different OVs using glioma cell lines and animal models, providing valuable data for the potential translation of these therapies to patients.

Novel oHSV generations were evaluated. NG34 is an ICP6 and ICP34.5-deleted virus, reengineered from rQNestin34.5 (Table 1) by switching out ICP34.5 with its human ortholog, GADD34, to restore the cytotoxic capacity of HSV. NG34 presented similar antitumor efficacy in immunocompromised and syngeneic GBM mouse models compared to rQNestin34.5, but NG34 appeared to be less toxic when injected in the brain of non-tumor-bearing mice [76]. In another study, Studebaker et al., evaluated the therapeutic potential of rRp450 using cell lines derived from medulloblastoma and atypical teratoid rhabdoid (AT/RT) tumors [77]. This virus carries a deletion in ICP6, retains ICP34.5, and expresses rat CYP2B1, an enzyme able to activate the chemotherapeutic prodrug cyclophosphamide (CP). Tumor-bearing mice treated with rRp450 displayed prolonged survival compared with vehicle control groups, with multiple complete responses, and the addition of CP-enhanced efficacy. This is in-line with a previous study supporting the hypothesis that oHSV can effectively infect and potently kill pediatric medulloblastoma [78]. Another interesting study reported that an oHSV-encoding human E-cadherin (OV-CDH1) protected virus-infected cells from lysis by natural killer (NK) cells [79]. This phenomenon facilitated the intratumoral virus spread, leading to the improved survival of OV-CDH1-treated GBM-bearing mice. Sette et al. also aimed at enhancing the intratumoral virus spread [80]. In this work, an epidermal growth factor receptor (EGFR)vIII-targeted/miR-124-sensitive oHSV [81] was engineered to express the matrix metalloproteinase (MMP) 9 protein. This virus is rendered tumor-selective based on recognition of the tumor-specific EGFRvIII mutant antigen and compromises synthesis of the essential ICP4 virus protein through the insertion of miR-124 recognition sites, a miR that is overexpressed in normal brain cells and absent in glioma cells. The expression of MMP9, specific for type IV collagen, a core component of the GBM extracellular matrix (ECM), showed an enhanced virus spread in neurospheres and improved survival of nude mice bearing GBM30 tumors.

The myxoma virus (MXVY) has not yet been evaluated in humans as an OV. However, this virus showed efficacy in preclinical GBM and medulloblastoma models, alone or in combination with rapamycin, among others, encouraging its translation to the clinic [82,83,84,85,86]. More recently, a M011L-deficient MYXV version, in which the viral antiapoptotic protein M011L was deleted, induced increased apoptosis in brain tumor-initiating cells, which are believed to mediate glioma recurrence [87]. Importantly, this treatment significantly prolonged survival in immunocompetent but not immunodeficient mouse models, suggesting that the antiviral immune response is essential to mediate the therapeutic efficacy of this virus.

Seneca Valley virus-001 (SVV-001) is a naturally occurring oncolytic picornavirus particularly attractive for brain tumors for its ability to cross the BBB upon systemic administration. This virus showed potent preclinical antitumor activity against medulloblastoma [88] and pediatric glioma [89] when administered intravenously. Both studies demonstrated that SVV-001 could efficiently cross the BBB barrier and eliminate pre-established tumors in vivo. Mechanistically, Liu et al., identified α2,3- and α2,6-sialic acids as key mediators of the SVV-001 infection of glioma cells [89]. Thus, the evaluation of α2,3- and α2,6-sialic acid tumor expression may become a diagnostic/prognostic factor when translated into clinical studies. Despite these promising preclinical results, no clinical study has been initiated to date.

Diverse engineered oncolytic VSVs have been designed to date for the treatment of brain tumors [90,91,92,93]. The most promising preclinical responses have been reported with the variant rVSV(GP) [94]. In this virus, the envelope glycoprotein (GP) of VSV, the key neurovirulence determinant, was replaced by the non-neurotropic lymphocytic choriomeningitis virus GP. This engineered virus caused no significant neurotoxicity upon administration into rodent brains and retained its potent oncolytic activity in both syngeneic and xenogeneic orthotopic brain cancer models. Remarkably, rVSV(GP) did not induce neutralizing antibody responses in mice, allowing repeated virus administrations. More recently, VSV GP was substituted for Ebola virus (EBOV) GP [95]. The resulting VSV-EBOV virus improved survival in tumor-bearing mice and showed a modest infection of normal brain cells.

Another virus worth mentioning due to its preclinical activity in glioma models is the Semliki forest virus (SFV). This virus has a natural neurotropism, making it an attractive candidate to treat neuroblastoma and GBM. Ramachandran and colleagues improved its safety by inserting target sequences for miR124, miR125, and miR134, miRNAs that are highly expressed in normal CNS cells but generally expressed at low levels in glioma cell lines, reducing its neurovirulence [96]. The resulting SFV4miRT virus showed an improved safety profile compared to its predecessor, SFV4.

The Zika virus (ZIKV) is known for its intrinsic ability to infect neural stem and progenitor cells during early development, causing brain abnormalities [97]. This virus is therefore a new promising therapeutic agent for malignant brain tumors. Zhu et al., reported that wild-type ZIKV preferentially targeted glioma stem cells (GSCs), as compared to differentiated glioma cells (DGCs) or normal neuronal cells [98]. In-line with this, another study corroborated the preferential oncolytic effect of ZIKV therapy in GSCs in vitro and the extended survival of tumor-bearing mice in a dose-dependent manner [99]. After these pivotal studies, Kaid et al. demonstrated a strong and specific oncolytic property of ZIKV against human CNS embryonal tumor neurospheres and in BALB/c mice bearing orthotopic human embryonal CNS tumor xenografts [100]. Longer survival, decreased tumor sizes, and complete responses were observed in vivo. The same group recently evaluated the feasibility of ZIKV treatment in two dogs bearing spontaneous intracranial tumors [101]. Tumor remission and the absence of adverse effects following intrathecal injections of ZIKV were observed. Taken together, these studies demonstrate the potential of ZIKV as oncolytic virotherapy in different CNS tumors. Nevertheless, applying this oncolytic virotherapy to patients requires wild-type ZIKV engineering to ensure reduced neurovirulence without affecting the oncolytic activity against GBM. Examples of such modifications are a live attenuated ZIKV vaccine (ZIKV-LAV) that contains a 10-nucleotide deletion in the 3′ untranslated region of the viral genome or a CpG-recoded ZIKV [102,103,104].

## 3. Combination Therapy in Preclinical and Clinical Settings

Malignant primary brain tumors remain devastating diseases, and immunotherapy has yet to be optimized to improve this. Evidence of the ability of OVs to convert a “cold” tumor microenvironment (TME) to an inflamed or “hot” tumor makes these agents attractive candidates to combine with other cancer therapies. Furthermore, despite the fact that OVs as single agents have shown indisputable promise for brain cancer therapy, events of striking tumor shrinkage in patients are still relatively rare. Therefore, combining OVs with other immuno- or chemotherapy agents is widely explored in order to achieve optimal tumor efficacy in patients with brain tumors, as discussed below.

### 3.1. Immune Checkpoint Inhibitors and Immunostimulatory Molecules

Many preclinical and clinical studies have shown that OVs enhance CD4+ and CD8+ T-cell tumor infiltration and increase the expression of PD-L1 in tumors. The benefits of combining OVs and checkpoint inhibitors have been reported in patients with diverse tumor types, with promising results [105,106]. Supporting the rationale of a combinational strategy, Hardcastle et al. demonstrated that the MV infection of GBM lines resulted in an initial increase of the immune evasion molecule PD-L1 [107]. Moreover, as the MV infection progressed, GBM cells produced and released DAMP molecules, such as HMB1 and HSP90. In vivo, a combination of MV and anti-PD-1 antibodies significantly increased the survival of mice bearing orthotopic GL261 GBM when compared to either agent alone. A similar approach was used in a study by Samson et al. [52], following their observation of immune cell infiltration and upregulation of the PD-1/PD-L1 axis upon the intravenous administration of reovirus in brain tumor patients. The treatment of C57/BL6 mice bearing GL261 tumors with a systemic infusion of a GM-CSF-expressing reovirus followed by anti-PD-1 antibodies resulted in improved survival as compared to single-agent therapies. In-line with this, Belcaid et al. recently demonstrated that low doses of the oncolytic adenovirus DNX-2401 (or Delta24-RGD) are sufficient to significantly alter the immune microenvironment in murine glioma, mostly by the upregulation of PD-1 and ICOS expression on CD8+ T cells [108]. Additionally, the brain tumor size was inversely correlated with the PD-1+ T-cell population in the tumor, suggesting that tumor regression is correlated with the local presence of PD-1+ T cells. Indeed, Delta24-RGD, in combination with anti-PD-1, significantly improved the overall survival in GL261 and CT2A orthotopic mouse models [108].

Beneficial combination therapy was also observed by Wirsching et al., who demonstrated that a miR-124-attenuated oHSV expressing ULBP3 promoted a localized and abscopal immune response, hence priming the TME for effective synergy with the anti-PD-1 checkpoint blockade in a GBM model [109].

Another slightly different strategy consists in modifying the virus to encode immune checkpoint antibodies. In a recent study, oHSV was engineered to express a single-chain fragment variable (scFv) against PD-1, named NG34scFvPD-1 [110]. Treatment with this virus showed the long-term survival of immunocompetent GBM-bearing mice and induced a memory immune response against the tumor, providing support of the combination of oHSV with a local expression of the PD-1 blockade. Another example is a study by Jian et al., who investigated the oncolytic Ad Delta-24-RGD expressing the immune costimulatory OX40L (Delta-24-RGDOX) [31]. Compared with its predecessor lacking the OX40L insert, Delta-24-RGDOX induced superior tumor-specific immunity in immunocompetent mice bearing GL261 tumors, which was not observed in immunodeficient mice. Furthermore, a combination of Delta-24-RGDOX with anti-PD-L1 induced the long-term survival of mice, with a high rate of complete tumor elimination.

Saha et al., exploited a triple-combination strategy [111,112]. They assessed the synergistic interaction between the IL-12-encoding oHSV (G47Δ-mIL12) and two checkpoint inhibitors, the anti-CTLA-4 and anti-PD-1 antibodies. Combining these three agents was necessary to overcome the immune-suppressive TME and cured a majority of the immunocompetent mice implanted with GBM GSCs. Remarkably, none of the cured rechallenged mice exhibited any pathological symptoms up to almost nine months post-treatment. Using a similar approach, a study showed that triple therapy using a VSV expressing the tumor-associated antigens HIF-2α, Sox-10, and c-Myc and anti-PD1/anti-CTLA-4 antibodies was the most effective as compared to VSV injection alone or in combination with either checkpoint blockade molecules alone [113]. Thus, these reports suggest that future studies may focus on multiple immunotherapeutic strategies in order to achieve improved antitumor efficacies.

Consistent with the therapeutic benefit of combining OVs with immune checkpoint inhibitors, a phase II clinical trial of a single intratumoral injection of DNX-2401 in combination with the anti-PD-1 antibody pembrolizumab in GBM patients is currently under investigation (NCT02798406). The interim analysis of this study revealed two partial responses and 100% survival at nine month for the first seven patients treated [70].

Finally, Tang et al. recently demonstrated the advantage of combining double-deleted vaccinia virus (vvDD) or MYXV expressing an IL15Rα-IL15 fusion protein with other treatments, including a vaccination with a GL261-specific neoantigen (GARC-1), rapamycin, celecoxib, and adoptive T-cell therapy [114]. The omission of any one of these therapies resulted in a greatly decreased treatment efficacy, whereas the combination resulted in the eradication of established GL261 glioma tumors in immunocompetent C57BL/6J.

### 3.2. CAR T-Cell Therapy

Chimeric antigen receptor (CAR) T cells have demonstrated impressive responses in hematological malignancies [115,116,117]. However, CAR T cells often find a strong immunosuppressive TME in solid tumors that prevents the T cells from exerting their full therapeutic potential (reviewed by [118,119]). OVs can potentially synergize with CAR T-cell therapy by altering the local TME immune system to further improve the T-cell proliferation, persistence, migration, and effector functions. Additionally, OVs can be engineered to express immunostimulatory molecules (e.g., chemokines, cytokines, and bispecific T-cell engagers) to better enhance the antitumor activity of CAR T cells. This strategy has been investigated in different solid tumor types, leading to the improved survival of tumor-bearing mice [120,121,122,123,124]. However, the synergistic effects of OVs and CAR T cells in the context of brain tumors remain to be tested. Recently, a study reported a deleterious effect of a murine interferon beta (IFNβ)-encoding VSV on EGFRvIII CAR T-cell efficacy against B16EGFRvIII tumors [125]. In this setting, only IFN-insensitive CAR T cells enabled a combinatorial therapy with VSVmIFNβ. This study highlighted the importance of understanding the immunobiology of such combinations to set the ground for further optimizations. In summary, OVs specifically designed to complement CAR T-cell effector functions would possibly improve therapy for brain tumors.

### 3.3. Standard Care—Chemotherapy and/or Radiotherapy

The standard of care for GBM includes maximal surgical resection followed by radiotherapy, plus concomitant and maintenance temozolomide (TMZ). Different OVs are currently under clinical evaluation, in combination with radiotherapy or chemotherapy (see Table 3). At the preclinical level, combination treatments between OVs and chemotherapy, mainly using TMZ, have been reported, showing synergistic activity and improved survival [126,127,128,129,130,131,132]. In-line with this, another recent study assessed the combined effects of the Delta-24-RGD virus with TMZ [133]. Interestingly, the combination regimen induced similar tumor CD8+ T-cell infiltration as compared to the virus alone in mice bearing intracranial GL261 tumors, but the infiltrating CD8+ T cells secreted more IFN-γ in response to tumor cells in Delta-24-RGD/TMZ-treated mice, improving the overall survival. In addition to a combination with TMZ, OVs able to efficiently convert the prodrug 5-FC into the cytotoxic agent 5-FU, such as the Toca 511 and TG6002 OVs, are under clinical evaluation (Table 2 and Table 3, respectively). Combinations of OVs with other pharmaceutical drugs have been also assessed. An example of that is the study by Lun et al., which showed enhanced viral replication and the prolonged survival of immunocompetent mice and rats injected with glioma cell lines when an intravenous delivery of vvDD-EGFP was combined with rapamycin or CP [134].

The synergy between OVs and radiotherapy has also been assessed. Recently, Martínez-Velez et al. demonstrated that combining the Delta-24-RGD virus with radiotherapy resulted in a synergistic antiglioma activity in vitro and in vivo in pediatric high-grade glioma (pHGG) and diffuse intrinsic pontine glioma (DIPG) models [135]. This combined therapy increased the tumor lymphocyte infiltration post-Delta-24-RGD injection, overcoming the immune “cold” status and potentially allowing the abscopal effect triggered by the radiotherapy to take place.

Rajaraman et al., showed that a sequential triple combination of TMZ or lomustine, oncolytic MV, and radiation therapy had a synergistic antitumor effect in glioma or stem-like glioma cell lines and identified a treatment-induced molecular and immunological signature [136], including an increase in chemokines regulating the immune response, an increase in the antigen presentation machinery, and in PD-L1 transcription. This study provides knowledge of immunological features that may help to improve combination treatments.

Another recent study demonstrated that oHSV-infected glioma cells induced the activation of NOTCH signaling in cancer cells adjacent to infected cells [137]. This pathway is frequently related to the increased survival of glioma cells, tumor progression, invasion, and therapy resistance [138]. Thus, the combination of oHSV with gamma secretase inhibitors (GSI) to block NOTCH signaling showed the sensitization of glioma cells to GSI after the oHSV treatment and increased survival of two different glioma models.

Overall, all these studies point out that the combination of OVs with chemotherapeutics or radiotherapy is additive and synergistic. However, to achieve the full potential, future studies should be addressed to determine the best selection of OV—and the transgenes carried—for each tumor, together with the best dose, route of delivery, and timing of each treatment. A deep understanding of these components is critical for a successful therapy.

## 4. Conclusions

As discussed in this review, several OVs have shown antitumor potential and good tolerance in varying scenarios and approaches, reinforcing the potential of these agents for the treatment of malignant brain tumors. However, there is still a long way to go for these therapies to get clinical approval as single agents. Tumor heterogeneity, physical barriers, antiviral immunity, immunosuppressive TME, and delivery are some of the main challenges for the success of oncolysis. Novel strategies need to be developed to overcome the aforementioned barriers and to improve the therapeutic potential of OVs (reviewed by [139]). Future research should be focused on answering what would be the most suitable virus type, delivery route, and doses for each case. On the other hand, the ability of OVs to attract T cells and to induce a durable immune response against both virus and tumor is broadly accepted. Thus, OVs can join forces with other cancer therapies, helping to overcome some of the obstacles due to the strong immunosuppressive mechanisms of these tumors. Nevertheless, to fully exploit the therapeutic potential of the synergistic effect, a better understanding of the patient-tumor immune status and the immunological features upon immunotherapeutic treatment is needed. Altogether, a strong understanding of these components will likely improve the treatment of these challenging tumors, OVs being relevant agents to impacting patient survival.

## Figures and Tables

**Table 1:** Summary of the main modifications of the oncolytic viruses evaluated in the clinic.

Virus Family	Name	Modification: Effect
**Adenovirus**	ONYX-015; DNX-2401; DNX-2440	E1B-55K deletion: selects tumors with deficient p53 E1AΔ24: selects cells with E2F-Rb deregulation RGD-4C insertion: facilitates viral entry to cells expressing αvβ3- and αvβ5-integrin OX40L insertion: increases T cell-mediated immunity
**Herpesvirus**	HSV1716; G207; G47Δ; C134; M032; rQNestin34.5	ICP34.5 deletion: makes it unable to replicate in normal neuronal cells ICP6 deletion: makes it unable to replicate in normal neuronal cells ICP47 deletion: prevents evasion from immune recognition IL-12 expression: immunostimulatory molecule
**Reovirus**	Reolysin	None
**NDV**	NDV-HJU; MTH-68/H	Attenuated strain: tumor-specific
**Poliovirus**	PVSRIPO	IRES replacement: eliminates neuropathogenesis
**Parvovirus**	ParvOryx01,	None
**Retrovirus**	Toca 511	*FCU1* insertion: encodes for an yCD enzyme able to locally convert the prodrug 5-FC to the drug 5-FU
**Measles virus**	MV-CEA; MV-NIS	Attenuated Edmonston strain: vaccine hCEA expression: to track the viral gene expression NIS expression: to track the viral gene expression and deliver radioactive iodine
**Vaccinia virus**	TG6002	*J2R* deletion: selects cells that express thymidine kinase *I4L* deletion: selects cells with ribonucleotide reductase subunits expression *FCU1* insertion: converts the prodrug 5-FC to the drug 5-FU

**Abbreviations:** hCEA: human carcinoembryonic antigen, IL-12: interleukin 12, IRES: internal ribosomal entry site, NIS: human thyroidal sodium iodide symporter, NDV: Newcastle disease virus, RGD: arginyl-glycil-aspartic acid motif, yCD: yeast cytidine deaminase, 5-FC: 5-Fluorocytosine, and 5-FU; 5-Fluorouracil.

**Table 2:** Completed oncolytic virotherapy clinical trials in patients with brain tumors.

Virus	Features	Phase	Disease	Delivery	Combination	Outcome	Reference
**Adenovirus**							
ONYX-015	E1B-55k and E3 deletion	I	Recurrent MG	IT		*n* = 24; PR: 1; PD: 23	[62]
DNX-2401	Δ24 deletion in E1A RGD-4C insertion	I	Recurrent MG	IT		*n* = 25; CR: 3; PR: 2; SD: 13; PD: 7	[12]
		I	Recurrent GBM	IT	TMZ	NA	NCT01956734
		Ib	Recurrent GBM or GSM	IT	IFN-γ	NA	NCT02197169
		I/II	GBM	IT		NA	NCT01582516
NSC-CRAd- Survivin-pk7	NSCs loaded with an OAd	I	MG	IT	Radiation TMZ	NA	NCT03072134
**Herpesvirus**							
HSV1716	ICP34.5 deletion	I	Recurrent MG	IT		*n* = 9; CR: 2; SD: 5; PD: 2	[63]
		I	MG	IT		*n* = 12; CR: 1; SD: 8; PD: 1	[64]
		I	MG	IT		*n* = 12; SD: 2; PD: 10	[65]
		I	Recurrent MG	IT	DXM	NA	NCT02031965
G207	ICP34.5 deletion ICP6 deletion	Ib/II	Recurrent MG	IT		*n* = 21; SD: 4; PD: 17	[66]
		Ib	Recurrent GBM	IT		NA	[67]
		I	MG	IT	5-Gy radiation	*n* = 9; PR: 1; SD: 5; PD: 3	[15]
G47∆	ICP34.5/ICP6/ICP47 deletion	I/IIa	Recurrent GBM	IT		NA	UMIN000002661
		II	Recurrent GBM	IT		NA	UMIN000015995
**Reovirus**							
Reolysin	Wild-type Serotype 3	I/II	Recurrent MG	IT		NA	NCT00528684
		I	Recurrent MG	IT		*n* = 15; PR: 1; SD: 10; PD: 4	[68]
		Ib	Brain tumor	IV		NA	[52]
**NDV**							
NDV-HJU	Attenuated NDV strain	I/II	Recurrent GBM	IV		*n* = 11; CR: 1; SD: 6; PD: 4	[53]
MTH-68/H		I	Advanced MG	IV		*n* = 12; PR: 4; SD: 3; PD: 5	[54,69]
**Parvovirus**							
ParvOryx01	Wild-type H-1	I/II	Primary or recurrent GBM	IT/IV		*n* = 18; SD: 6; PD: 12	[51]
**Retrovirus**							
Toca 511	Nonlytic *FCU1* insertion	I	Recurrent MG	IT/IV	5-FC prodrug	*n* = 43; CR: 2; PR: 2; SD: 8; PD: 31	[14,60]
**Measles virus**							
MV-CEA	Attenuated hCEA expression	I	Recurrent GBM	IT		*n* = 22; SD: 20; PD: 2	NCT00390299

**Abbreviations:** AA: astrocytoma, CR: complete response, DXM: dexamethasone, 5-FC: 5-Fluorocytosine, GBM: glioblastoma, GSM: gliosarcoma, hCEA: human carcinoembryonic antigen, IFN-γ: interferon gamma, IT: intratumorally, IV: intravenously, MG: malignant glioma, *n*: number of patients enrolled, NA: information not available, NDV: Newcastle disease virus, NSC: neural stem cell, OAd: oncolytic adenovirus, PD: progression disease, PR: partial response, RGD: arginyl-glycil-aspartic acid motif, SD: stable disease, and TMZ: temozolomide.

**Table 3:** Current active oncolytic virotherapy clinical trials in patients with brain tumors.

Virus	Features	Phase	Disease	Delivery	Combination	Reference
**Adenovirus**						
DNX-2401	Δ24 deletion in E1A RGD-4C insertion	II	BT	IT	Pembro	NCT02798406 [70]
		I	Naïve DIPG	IT		NCT03178032 [29]
DNX-2440	DNX-2401 with OX40L expression	I	Recurrent GBM	IT		NCT03714334
**Herpesvirus**						
G207	ICP34.5 deletion ICP6 deletion	I	Recurrent supratentorial BT	IT	-/5-Gy radiation	NCT02457845
		I	Recurrent cerebellar BT	IT	-/5-Gy radiation	NCT03911388
G47∆	ICP34.5 and ICP47 deletion ICP6 deletion	I	Olfactory NBM	IT		UMIN000011636
C134	ICP34.5 deletion Expression of IRS1	I	Recurrent MG	IT		NCT03657576
M032	ICP34.5 deletion IL-12 expression	I	Recurrent GBM, AA or GSM	IT		NCT02062827 [71]
rQNestin34.5	ICP34.5 expression under nestin promoter	I	Recurrent MG	IT	CP	NCT03152318
**Reovirus**						
Reolysin	Wild-type Serotype 3	I	Pediatric relapsed BT	IV	GM-CSF	NCT02444546
**Poliovirus**						
PVSRIPO	Attenuated IRES replaced by IRES from HRV2	I	Recurrent MG	IT		NCT01491893 [13]
		Ib	Pediatric recurrent MG	IT		NCT03043391
		II	Recurrent MG	IT		NCT02986178
**Vaccinia virus**						
TG6002	*J2R/ I4L* deletions *FCU1* insertion	I/IIa	Recurrent GBM	IV	5-FC prodrug	NCT03294486
**Measles virus**						
MV-NIS	Attenuated strain Expression of NIS	I	Recurrent MB or AT/RT	IT		NCT02962167

**Abbreviations:** AA: astrocytoma, AT/RT: atypical teratoid rhabdoid tumor, BT; brain tumor, CP: cyclophosphamide, 5-FC: 5-Fluorocytosine, GBM: glioblastoma, DIPG: diffuse intrinsic pontine glioma, GM-CSF: granulocyte-macrophage colony-stimulating factor, GSM: gliosarcoma, IRES: internal ribosomal entry site, IT: intratumorally, IV: intravenously, MB: medulloblastoma, MG: malignant glioma, NBM: neuroblastoma, NIS: human thyroidal sodium iodide symporter, Pembro: pembrolizumab, and RGD: arginyl-glycil-aspartic acid motif.
